# Characterization and Analysis of Metal Adhesion to Parylene Polymer Substrate Using Scotch Tape Test for Peripheral Neural Probe

**DOI:** 10.3390/mi11060605

**Published:** 2020-06-22

**Authors:** Seonho Seok, HyungDal Park, Jinseok Kim

**Affiliations:** 1 Center for Nanoscience and Nanotechnology (C2N), University-Paris-Saclay, Orsay 91400, France; seonho.seok@u-psud.fr; 2 Center for Bionics, Korea Institute of Science and Technology (KIST), Seongbuk-gu, Seoul 02792, Korea; hyungdal@kist.re.kr

**Keywords:** adhesion, thin film metal, parylene, neural probe, scotch tape test, FEM

## Abstract

This paper presents measurement and FEM (Finite Element Method) analysis of metal adhesion force to a parylene substrate for implantable neural probe. A test device composed of 300 nm-thick gold and 30 nm-thick titanium metal electrodes on top of parylene substrate was prepared. The metal electrodes suffer from delamination during wet metal patterning process; thus, CF_4_ plasma treatment was applied to the parylene substrate before metal deposition. The two thin film metal layers were deposited by e-beam evaporation process. Metal electrodes had 200 μm in width, 300 μm spacing between the metal lines, and 5 mm length as the neural probe. Adhesion force of the metal lines to parylene substrate was measured with scotch tape test. Angle between the scotch tape and the test device substrate changed from 60° to 90° during characterization. Force exerted the scotch tape was recorded as the function of displacement of the scotch tape. It was found that a peak was created in measured force-displacement curve due to metal delamination. Metal adhesion was estimated 1.3 J/m^2^ by referring to the force peak and metal width at the force-displacement curve. Besides, the scotch tape test was simulated to comprehend delamination behavior of the test through FEM modeling.

## 1. Introduction

Many research efforts have been made to develop and improve of the prosthetic hands and arms for the amputees, and, in recent years, much progress has been observed in the development of life-like robotic hands and the means of controlling them with greater degree of freedom. For this purpose, micro-electro-mechanical systems (MEMS) technologies have been used to fabricate neural interface probes [1,2,3,4,5,6]. However, existing MEMS-based neural electrodes would have a limitation on the neural interface due to its material characteristics. Thus, flexible neural electrodes have been recently proposed to minimize mechanical mismatch between the electrode and tissue after the electrode’s implantation for a stable long-term recording and stimulation. To this sense, peripheral neural interface (PNI) devices have appeared to retrieve and send neural signals directly from and to the residual or existing peripheral nerves in this field [7]. Recently, thin film flexible polymeric devices are being used for measuring nerve impulse from the central or peripheral nerve systems [8,9,10,11,12,13]. Such flexible polymeric devices tend to be designed several μm in thickness and a few mm in length due to its nature of interfacing with neurons in human body. Consequently, metal electrodes on such a thin and long polymeric substrate have constraints to be at best several hundred nanometers. Thus, metallization technology frequently used is PVD (Physical Vapor Deposition), such as evaporation or sputtering. The PVD metal layers are formed into long and thin metal lines through wet-etch or lift-off technique. Although thin-film polymeric devices are flexible and biocompatible, they are prone to delamination and carry concerns about their mechanical robustness [14]. A nanopillar array created by plasma etching could be used to enhance adhesion among different materials in the parylene-metal-parylene system [15]. ALD (Atomic layer deposition) Al2O3 combined with the silane adhesion promoter A-174 would increase adhesion force between two parylene films [14]. Parylene material has been shown that mechanical properties can be maintained after stored in PBS (Phosphate Buffered Saline) solution up to 12 months [16]. Therefore, mechanical or chemical treatments of the interface between polymer substrate and metal film is frequently required [17]. However, concrete characterization of metal adhesion to parylene substrate dedicated to neural probe is still insufficient. Scotch tape test has been frequently used to estimate adhesion force of metal layers to polymer substrates [18,19,20]. Tape is often used to overcome the adhesion challenges in what’s commonly known as the “tape test,” which is a variation of ASTM (American Society for Testing and Materials) D-3359 [21].

In this paper, metal adhesion to parylene substrate has been characterized by using scotch-tape test and it has been analyzed based on Finite Element Method (FEM) modeling. Sample preparation for adhesion test is presented in Section 2. Scotch tape test results are explained in Section 3. FEM modeling and simulation of the scotch tape test is depicted in Section 4. Finally, conclusion will be made in Section 5.

## 2. Test Sample Preparation for Metal Parylene Adhesion Test

**Figure 1:**
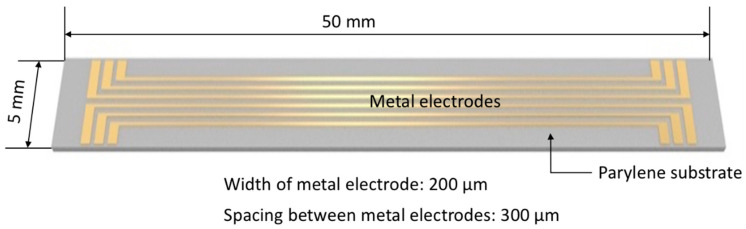
Design of test pattern.

**Figure 2:**
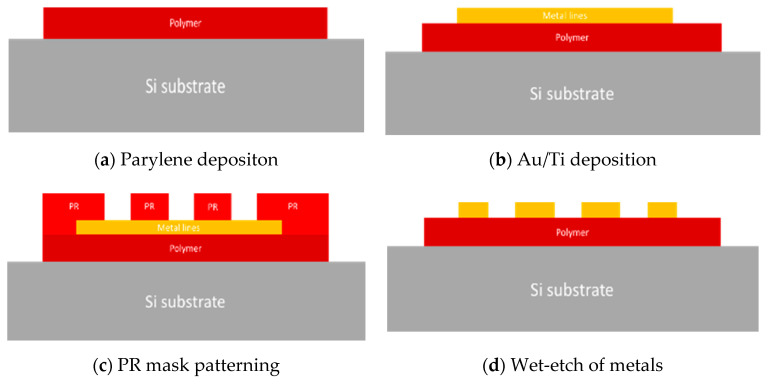
Test sample fabrication process.

**Figure 3:**
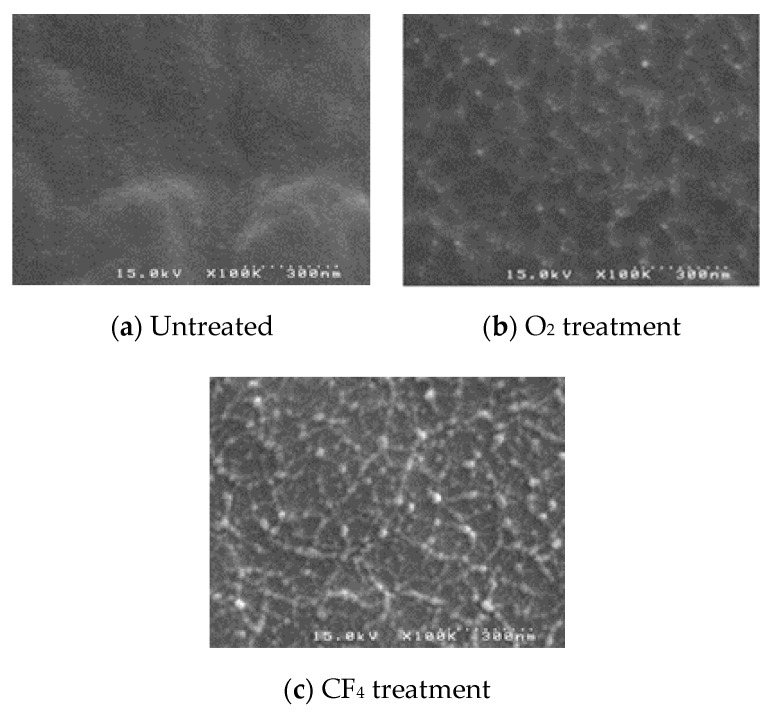
Parylene surface modification.

Figure 1 shows the schematic of test pattern; it has length of 50 mm and width of 5 mm. It consists of 6 metal lines; each metal line has 200 µm in width and is spaced at 300 µm as is the design of multi-channel neural probe. Test pattern fabrication process is shown in Figure 2; (a) A parylene (parylene-C) layer having 5 µm in thickness was deposited in a 4-inch Si wafer using by commercial parylene coater system (VPC-500, Paco Engineering, Incheon, Korea). The monomer was deposited on the surface of the silicon wafer at a vapor phase condition with 0.8 μm/min, and deposition temperature was 20 °C. (b) Before metal layers deposition, the parylene surface was etched with CF_4_ gas (process conditions; (25 mTorr, 20 sccm, 1.5 min)) without O_2_ in order to increase surface roughness reducing the hydrophobicity, as shown in Figure 3. Note that O_2_ was not used for surface modification since the parylene is susceptible to be surface oxidation causing degradation of mechanical properties of the polymer neural probe. Moreover, the nanopillar structures on parylene substrate was efficiently built with CF_4_ gas rather than O_2_ gas, as shown in Figure 3b,c. Besides, there have been two major methods to improve interfacial adhesion between parylene and metal layer: chemical treatment or mechanical surface modification with RIE (Reactive Ion Etching) etch (Plasma-therm 790 MF, Plasma-Therm, Saint Petersburg, FL, USA). Concerning the test sample used in our study, parylene was already deposited on a silicon substrate; thus, chemical treatment may have changed the properties of parylene substrate itself, as well as the interface on which the metal was deposited. Furthermore, RIE etch have shown better performance compared with conventional A-174 saline chemical treatment [15]. Therefore, we modified the parylene surface with CF_4_ plasma etch to make a nanopillared surface, increasing the interfacial energy. The effectiveness of the nanopillared parylene surface was confirmed during metal patterning step. Titanium (30 nm) and gold (300 nm) were sequentially deposited with using by E-beam evaporator (ei5, ULVAC, Methuen MA, USA) on the parylene substrate without rupture of vacuum. (c) Photoresist (AZ GXR 601 (46cp), Merck, Kenilworth, NJ, USA) was patterned as a metal etch mask. The process conditions are summarized in Table 1. (d) Gold was first etched with Au etchant (Gold ETCH TFA, Transene Company, Danvers, MA, USA) for 5 min, and then titanium is etched with BOE (Buffered Oxide Etchant) solution (Buffered oxide etch 6:1, VWR International, Radnor, PA, USA) for 10 s. After metal patterning, PR mask was removed with acetone. Etch time was 60 s.

**Figure 4:**
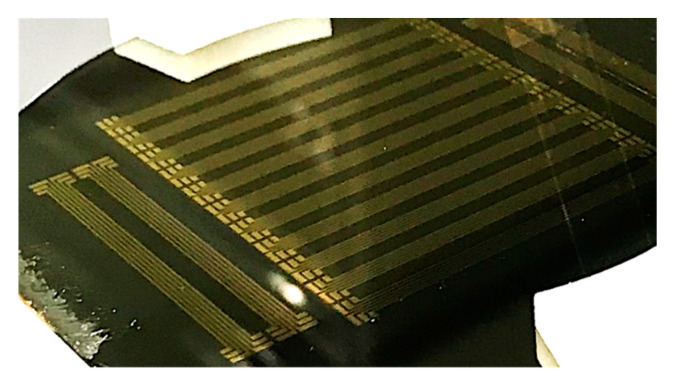
Test samples fabricated on 4-inch silicon substrate.

Resultant test samples on 4-inch silicon wafer is shown in Figure 4. All of metal lines were successfully implemented on the parylene substrate without any delamination during etching process. Note that the metal lines were fully delaminated from the parylene substrate during metal wet-etch step.

## 3. Scotch Tape Test for Metal Adhesion to Parylene Substrate

**Figure 5:**
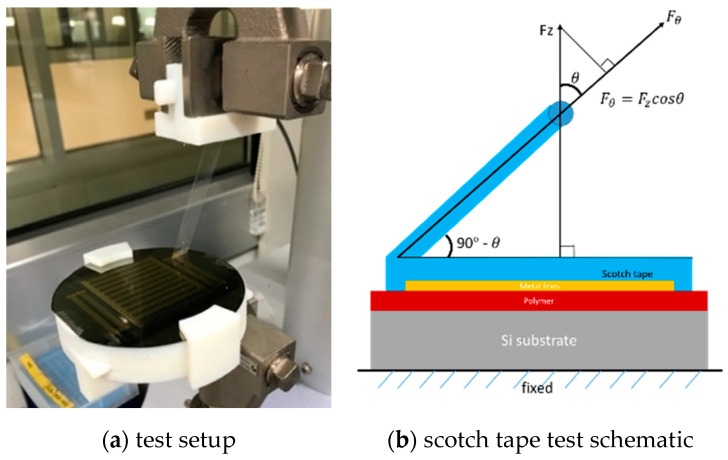
Scotch tape test for metal adhesion to parylene substrates.

The prepared test samples underwent the scotch tape test to evaluate adhesion strength of the metal electrodes to the parylene substrate. The machine used for the scotch tape test was Shimadzu EZ-S machine (Shimadzu, Kyoto, Japan) dedicated for tensile testing. Figure 5 shows a photo of scotch tape attached on the test sample and schematics of the scotch tape test, respectively. The scotch tape is 3 M transparent tape 550. It has thickness of 50 μm and 12 mm width, and it provides adhesion to steel of 1.8 N/cm (or 0.18 N/mm). 

Referring to Figure 5b, the scotch tape test was carried out in the following way; the machine applies stroke (unit: mm/min) into one end of the scotch tape, and then it measures force *F_z_* (unit: N). As force of interest is *F_θ_*, relationship between *F_z_* and *F_θ_* can be calculated as Equation (1).

(1) $$F_{\theta }=F_{z}cos\;\theta .$$

During the scotch tape test, the angle (90°−*θ*) was changed from 60° to 90°; thus, *F_θ_* = 0.5 *F_z_* at 45°, and *F_θ_* = *F_z_* at 90°. For simplicity, we used the measured *F_z_* from the scotch tape test to extract the adhesion strength.

**Figure 6:**
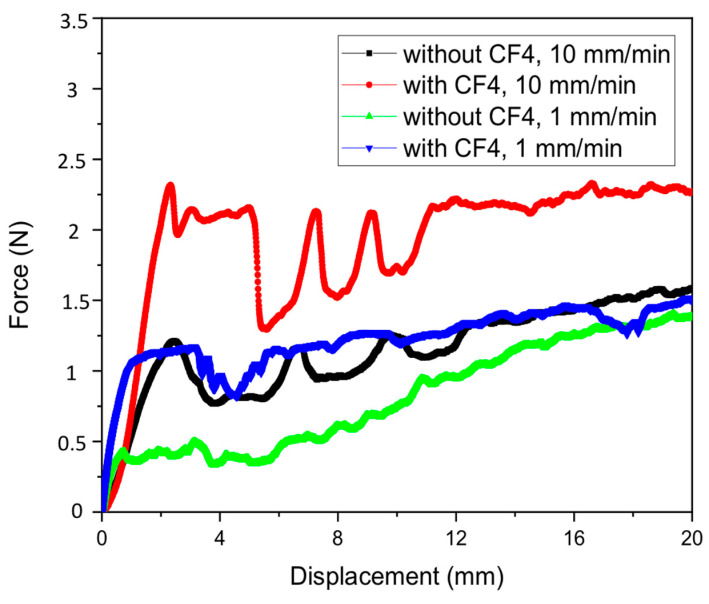
Force-displacement curves from scotch tape test with metal lines on parylene substrate.

Metal adhesion to parylene substrate was then measured with the scotch tape test. Scotch tape was attached to the parylene surface, slightly away from the left-end metal line to the right-end of the metal. After that, two different strokes (10 mm/min and 1 mm/min) were applied to the scotch tape, and corresponding force was measured as shown in Figure 6. All metal lines were debonded from the parylene substrate for all the cases. It was found that the sample with CF_4_ treatment needs more force than that without CF_4_ treatment, which means a parylene surface with CF_4_ treatment sticks better to scotch tape. This is a proof that parylene surface energy can be increased with only CF_4_ treatment without O_2_. 

**Figure 7:**
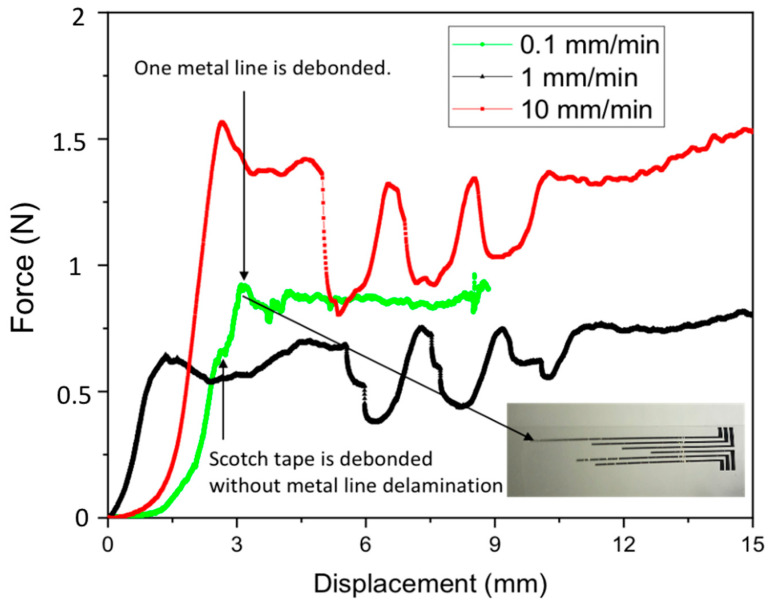
Force-displacement curves from scotch tape test with metal lines on parylene substrate with CF_4_ treatment.

The first peak in each measured force was due to initiation of metal debonding, which makes abrupt drop of force. Minimal adhesion force is found when a stroke of 1 mm/min was applied to the parylene test sample without CF_4_ treatment (green line). It can be said that the metal adhesion had lower than the adhesion value estimated from the first peak. The adhesion can be calculated as follows; (0.5 N/12 mm) × (1.2 mm/12 mm) = 4.2 N/m. As all metal lines were debonded, the metal adhesion should have had lower than 4.2 J/m^2^. Thus, a lower stoke of 0.1 mm/min was applied to find metal adhesion to the parylene substrate. In this case, the scotch tape was attached only to the narrow metal lines of CF_4_ treated parylene and then force was recoded while stroke of 0.1 mm/min is applied. The measured force-displacement curve was compared with the previous results of 1 mm/min and 10 mm/min, as shown in Figure 7. As remarked in Figure 7, the scotch tape was debonded up to 0.9 N without metal line delamination, and one metal line started to debond from 0.91 N. Therefore, metal adhesion could be extracted from this peak force; (0.91 N/12 mm) × (0.2 mm/12 mm) = (76 N/m) × (0.017) = 1.29 N/m = 1.29 J/m^2^. Note that inset shows transferred metal lines on the scotch tape. Scotch tape strokes of 1 mm/min and 10 mm/min introduced large force fluctuation, which would result from relatively large applied force compared with interfacial energy. 

Table 2 summarizes adhesion force between parylene and metal layer of previous reports compared with this work. The majority of the studies on metal-parylene adhesion force deal with parylene layer deposited on metal film with inteface treatment. Thus, they can provide from tens N/m to hundreds N/m of adhesion force. A thin metal layer, like Al thin film, on PET(Polyethylene terephthalate) layer has 46.7 N/m of adhesion. The thin gold metal line of this work had 1.29 N/m of adhesion force, which may result from the relatively narrow metal line of 200 μm.

## 4. FEM Modeling and Simulation

FEM modeling and simulation is very useful to understand stress effect and corresponding deformation of MEMS package, debonding characteristics of a transfer packaging, and mechanical behaviors related with delamination [23,24,25,26,27]. Especially, debonding of a substrate and film delamination can be studied by adopting a CZM (Cohesive Zone model) to represent the interface of interest [28,29,30].

**Figure 8:**
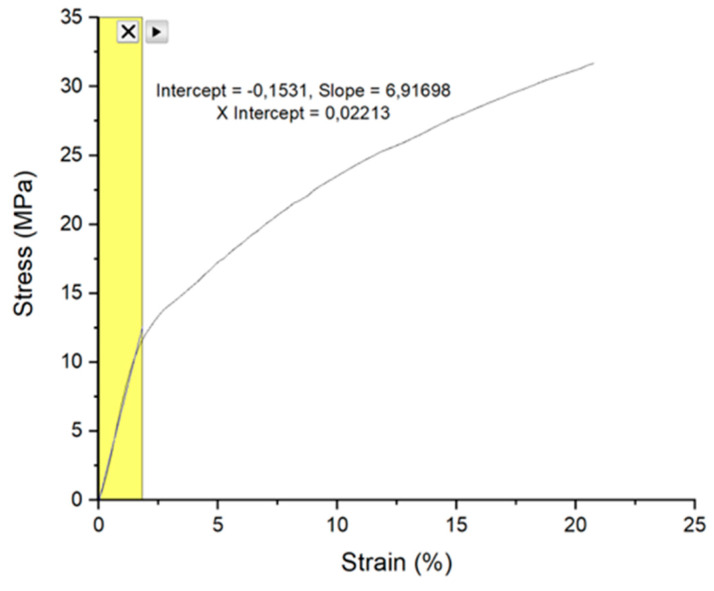
Tensile test result of scotch tape.

For FEM modeling, material properties of each element are important to get good simulation results. Required material properties in this modeling are Young’s moduli and poisson ratios of scotch tape and parylene and strain energy release rate of interface between the scotch tape and parylene. Young’s modulus of the scotch tape is extracted from tensile test result, as shown in Figure 8. The Young’s modulus of the scotch tape is 6.9 MPa in the elastic region, and the maximum applied force in the elastic region is 7.6 N when the applied strain is 2.2% (2.2 mm elongation as test scotch tape length is 100 mm). From the tensile test result, scotch tape in the metal adhesion test would be in the elastic region as the applied force is less than 2 N in all the cases. Poisson ratio of the scotch tape is assumed to be 0.4 as other polymer materials. Young’s modulus and poisson ratio of parylene are 2.67 GPa, as extracted in previous work, and 0.4, respectively [23]. Table 3 summarizes material properties for the FEM model. Note that interface material properties were defined with critical strain energy release rate. The value for critical strain energy release rate was the measured adhesion force as explained earlier.

Given with material properties, modeling and simulation of the scotch tape test was performed in two steps: 1) crack propagation behavior of the interface between the scotch tape and parylene substrate 2) debonding of the scotch tape from parylene substrate based on CZM.

**Figure 9:**
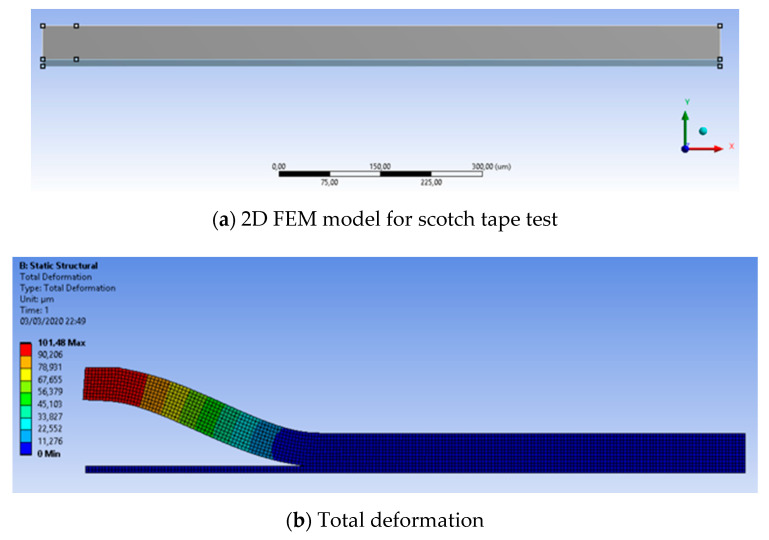

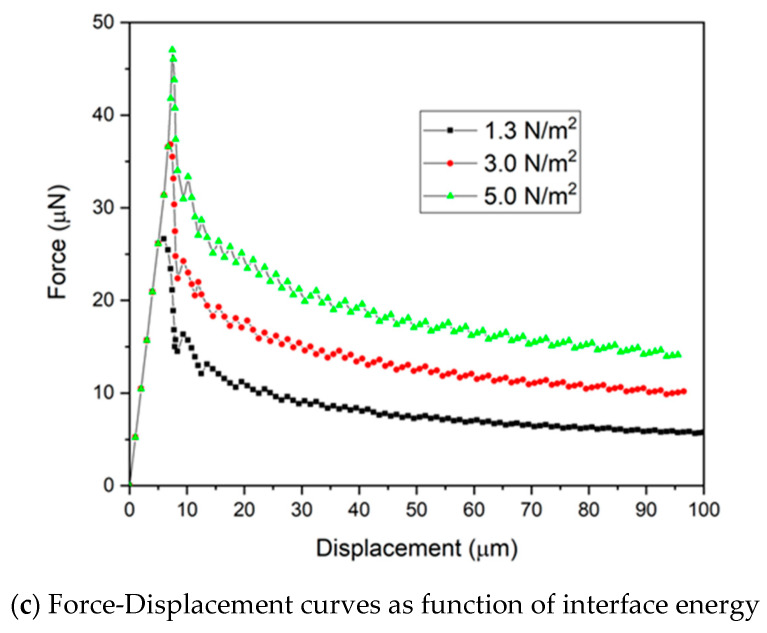
Crack propagation model and simulation results.

A 2D FEM model for crack propagation was built, as shown in Figure 9a. The length of this model was 1000 µm, and thickness was 50 µm for scotch tape and 5 µm for parylene polymer. The following boundary conditions were applied: bottom line is fixed and displacement load is applied to left-top end, having 50 µm width. Note that 2D model behavior was defined as plane strain. Total deformation of the model when displacement of 100 μm in y direction was applied to the scotch tape is presented in Figure 9b. As is in the scotch tape test, delamination of the interface between scotch tape and parylene occurred, and crack propagated in x-direction.

Force-displacement was investigated as function of interface adhesion energy, as shown in Figure 9c. The required force for crack initiation was increased as interface adhesion energy increased, as expected. The force magnitude smaller than the measurement would have been due to thickness effect in 2D simulation. An important parameter in this graph is minimal displacements for crack initiation: 5 µm, 6.7 μm, and 7.6 μm for 1.3 N/m^2^, 3.0 N/m^2^, and 5.0 N/m^2^, respectively. These parameters are included in the following 3D interface delamination as a part of CZM parameters. From the simulation results, strain energy release rate (SERR) for mode 1 referring to VCCT (Virtual Crack Closure Technique) (G1), SERR for mode 2 from VCCT (G2), and SERR for mode 3 from VCCT (G3) were found 0.5 J/m^2^, 0.75 J/m^2^ and 0 J/m^2^, respectively. The total amount of VCCTs corresponded to the interface energy of 1.3 N/m^2^. Principal modes of the fracture of the delamination was from mode 1 and mode 2.

**Figure 10:**
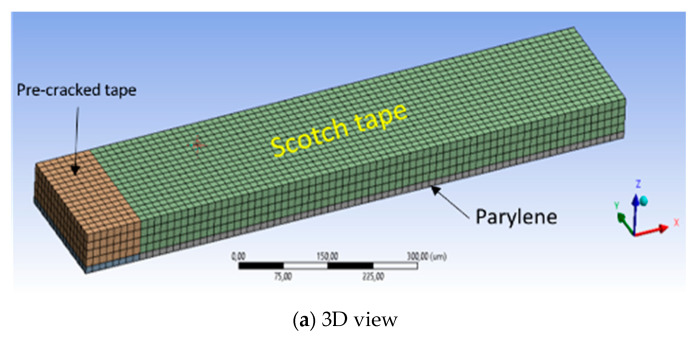

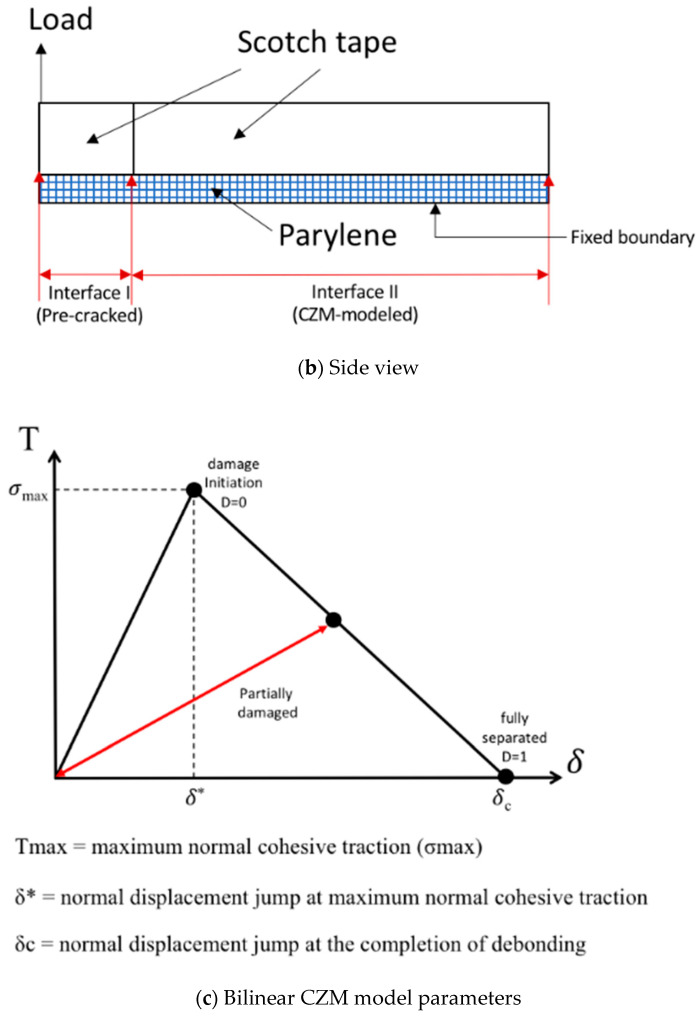
Three-dimensional Finite Element Method (FEM) model for Cohesive Zone model (CZM) interface delamination.

Figure 10a shows 3D model for the CZM interface delamination. As indicated in Figure 10b, there were two different regions in this model: pre-cracked (interface I) and CZM-modeled (interface II). CZM is a useful way to simulate interface delamination, which is frequently used for thin film delamination and transfer packaging technique [24,25]. The interface II, which is of interest for the adhesion, is modeled with CZM (Cohesive Zone Model) parameters, as explained in Figure 10c. As the bilinear CZM model needs at least two parameters, maximum normal traction and normal displacement at debonding was defined, as presented in Table 4. The minimal gaps for the fracture initiation found from the previous crack propagation simulation were included for 3D CZM simulation to estimate applied force to initiate the interface crack. 

**Figure 11:**
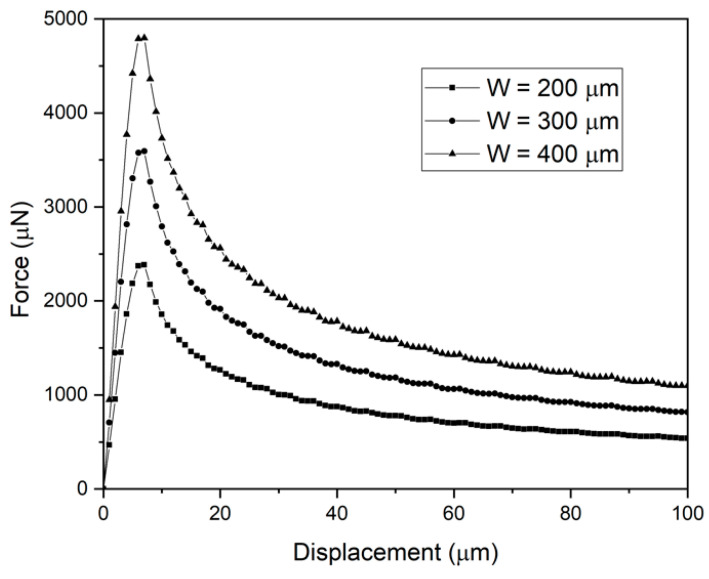
Force-displacement curves as function of metal width.

A displacement load was applied to one-end of the scotch tape, and then the force-displacement was extracted from the simulation. Referring to bilinear CZM model, critical strain energy release rate was calculated 1.25 J/m^2^. Initial width of the 3D model was 200 µm as was the fabricated metal electrode width. As in the 2D case, displacement load was applied to left-tip end. Extracted force-displacement curve at the loading place is presented in Figure 11. Minimal force for debonding of scotch tape was estimated 1.2 N/m, while measured one was 1.29 N/m. Adhesion force of the simulation had a good agreement with the measurement. Width of metal electrode could have been increased to get larger interface adhesion, as shown in Figure 11 When wider metal electrode is used to achieve larger metal-parylene adhesion, metal line impedance for neural signal acquisition should be taken into account.

## 5. Conclusions

Thin film flexible polymeric devices, such as a parylene-metal-parylene system, are being used for measuring nerve impulse from the central or peripheral nerve systems. Such thin-film polymeric devices provide advantages of flexibility and biocompatibility, but they are prone to delamination and carry concerns about their mechanical robustness. Therefore, metal adhesion strength to polymer substrate is important. The adhesion of metal electrodes to parylene substrate was measured by the scotch tape test. Thin and long metal electrodes was patterned on a parylene substrate in which the surface was modified by CF_4_ plasma etch before the metal deposition through e-beam evaporation. Metal adhesion strength was estimated by measuring force-displacement curve of the scotch tape test. The estimated metal adhesion was 1.3 J/m^2^. Experiment result was verified through FEM modeling of the scotch tape test. The proposed modeling method provided adhesion force having good agreement with experimental result. Although a thin-film parylene-based device can provide excellent short-term reliability, there exists one significant drawback of poor adhesion to metallic layer. The failure of the metal electrode on the parylene substrate is accelerated in the wet environment of a human body and under mechanical forces originating from body movement. Therefore, mechanical integrity in conditions of a human body implant or movement will be performed to assess long-term reliability of the parylene-metal devices, along with the biocompatibility of the parylene-base neural probe.

## Figures and Tables

**Table 1:** Photoresist etch mask process conditions.

Step	Conditions
PR coating	10 s @1500 rpm
Soft bake	60 s @ 95 °C
Exposure	10 s @10 mW
Develop	10 s @developer
Post exposure bake	30 s @110 °C
Thickness	1.2 μm

**Table 2:** Comparison of other research results with scotch tape test.

Test Method	Test Sample Structure	Interface Treatment	Environment	Remarks
		Adhesion Force		
Peel 90° [18]	Parylene (10 μm)/sputtered Ti (up to 100 nm)/Au (300 nm)/glass substrate	Gorham process before parylene deposition to make adhesion promoting layer	48 h in PBS solution	metal width = 10 mm
		35.5 mN/mm		
Peel 180° [19]	Al thermal evaporated metal (60 nm)/PET (12 μm)/Al substrate (1 mm)	-	RT	metal width = 15 mm
		0.7 N/15 mm		
Tensile [15]	Au (200 nm)/Cr (13 nm)/Parylene (10 μm)/glass substrate	O_2_ RIE etching	RT	metal width = 15 mm
		2.13 ± 0.12 MPa		
Peel test [22]	Parylene (9–20 μm)/Ti substrate	Plasma Polymerized Ethane (PPE)	RT	metal width = NA
		0.34 N/mm		
Peel test [This work]	Au(300 nm)/Ti(30 nm)/Parylene(5 μm)/Si substrate	CF_4_ RIE	RT	metal width = 200 μm
		1.29 N/m		

**Table 3:** Material properties of materials and interface.

Material Properties	Scotch Tape	Parylene
Young’s modulus (Pa)	6.9 × 10^6^	2.7 × 10^9^
Poisson ratio	0.4	0.4
-	Interface	
Critical mode I energy release rate (J/m^2^)	1.3	
Critical mode II energy release rate (J/m^2^)	1.3	
Critical mode III energy release rate (J/m^2^)	1.3	

**Table 4:** Cohesive Zone model (CZM) parameters.

Parameter Name	Value
Maximum normal traction	0.5 MPa
Normal displacement jump at completion of debonding	5 µm
Maximum tangential traction	0.5 MPa
Tangential displacement jump at completion of debonding	5 µm
